# Palliative Care Environments in an Acute Hospital for End‐Of‐Life Care—Perspectives of Patients, Next of Kin and Healthcare Professionals

**DOI:** 10.1002/nop2.70225

**Published:** 2025-04-17

**Authors:** Dorte Buchwald, Cecilie Hübner, Louise Sofia Madsen, Dorte Melgaard

**Affiliations:** ^1^ Palliative Care Team, North Denmark Regional Hospital Hjoerring Denmark; ^2^ Department of Surgery North Denmark Regional Hospital Hjoerring Denmark; ^3^ DEFACTUM, Central Denmark Region Århus Denmark; ^4^ Department of Clinical Medicine Aalborg University Aalborg Denmark; ^5^ Department of Acute Medicine and Trauma Care Aalborg University Hospital Aalborg Denmark

**Keywords:** design, family, healing architecture, palliation, patient‐/person‐centred care

## Abstract

**Aim:**

The aim of this study is to explore the perspectives of patients, next of kin and healthcare professionals on planning a new end‐of‐life room in a department of surgery in an acute hospital.

**Design:**

Qualitative study.

**Methods:**

Between January and March 2023, 20 individual semi‐structured interviews were conducted at the REDACTED. Participants included seven seriously ill and dying patients, three next of a kin and ten health care professionals. The interviews focused on identifying key elements that participants deemed important in an end‐of‐life room during the final days of life.

**Findings:**

Data analysis revealed two main themes: ‘Lively physical surroundings’ and ‘Human and medical competencies’ of the health care professionals. The findings highlight a shared understanding among participants of the importance of lively physical surroundings, including aesthetics, connections to nature, appropriate lighting and the creation of a homely atmosphere.

**Patient or Public Contribution:**

Although patients and next of kin were not involved in the planning of the study due to ethical considerations, nurses working on the ward contributed valuable insights during the study's design. Their input helped ensure the relevance and feasibility of the research in a clinical setting.

**Reporting Method:**

COREQ was used as aguideline.

## 1 Introduction

The significance of palliative care environment design on patient outcomes is particularly pronounced for individuals receiving palliative care. Historical figures like Marcus Vitruvius Pollio, Hippocrates and Mother Teresa recognised the profound link between human well‐being, health and the surrounding environment. With the evolution of healing architecture, modern healthcare facilities increasingly integrate these insights into their building design and patient care practices (Badash et al. 2017; Thaddeus and Napoleon 2021).

Numerous studies have highlighted specific components of the physical environment that can alleviate suffering and enhance the quality of life for end‐of‐life patients and their families (Sagha Zadeh et al. 2017). Zadeh et al.'s (Sagha Zadeh et al. 2017) comprehensive review identifies key factors affecting end‐of‐life care, including social interaction, positive distractions, privacy, personalization, creating a home‐like environment and establishing an ambient atmosphere. Additionally, Wong et al.'s (Wong et al. 2023) narrative review supports these factors while emphasising the importance of a connection to nature. Confirming the findings of previous reviews, Brereton et al. (Brereton et al. 2012) present a study delineating crucial elements for end‐of‐life care for older adults and their families. Rowlands and Noble's (Rowlands and Noble 2008) study underscores similar conclusions while emphasising the significance of effective staff communication in reducing anxiety and improving outcomes for both patients and their families. All the studies were conducted by researchers rooted in and influenced by the palliative care culture. Studies document that the preferences of patients and their families for the environment during their final moments are crucially important. When healthcare professionals were asked about their perspectives on the ideal physical environment for end‐of‐life care in acute hospital settings, their views largely aligned with those of patients and next of kin. They emphasised the need for a homely environment, an optimised physical setting and considerations for the patients' families (Gardiner et al. 2011; McLaughlan et al. 2022).

In Denmark, at the REDACTED, a decision has been made to create an end‐of‐life room within the surgery department to better meet the needs of patients nearing the end of their lives while receiving care in the department. Traditionally, there are cultural differences between medical specialties, including surgical and palliative care. Therefore, it is relevant to explore how the design of the new rooms should accommodate the needs of both dying patients and their relatives, as well as the staff. To ensure the best possible environment, it is crucial to consider and merge the complex needs of both patients, next of kin and staff when planning new end‐of‐life rooms in an emergency hospital.

This study aimed to explore the perspectives of patients with palliative needs, next of a kin and healthcare professionals on how to plan a new end‐of‐life room in a department of surgery in an acute hospital.

### 1.1 Materials and Methods

To obtain transparency, this study was reported following the Consolidated Criteria for Reporting Qualitative Research as a reporting guideline (Tong et al. 2007).

### 1.2 Methodology and Theory

The methodological approach chosen for this study was Interpretive Description, aiming to establish practice‐based contextualised knowledge to identify the perspectives of patients, next of kin and health care professionals through semi‐structured individual interviews (Thorne 2016). The inductive nature of Interpretive Description allows for a deep exploration of the perspectives of patients, next of kin and healthcare professionals. This is essential in understanding the multifaceted needs and preferences in an end‐of‐life context, as these perspectives are deeply personal and context‐specific. Interpretive Description focuses on generating actionable, practice‐based knowledge rather than purely theoretical insights, and this aligns perfectly with the study's aim as the findings can directly inform practical changes in healthcare environments.

For individuals at the end of life, they find themselves in a unique situation. In this context, Antonovsky's (Antonovsky 1987) theoretical perspective becomes valuable in understanding how they navigate and cope with this situation. The study's premise is that people's sense of coherence reflects their confidence in the predictability of life events and their capacity to adapt to new circumstances that arise in the end of their life therefore Antonovsky's concept of sense of coherence was used to understand the statements of the participants. Sense of coherence represents an individual's orientation towards life or in this case death, expressing the degree of confidence they have in understanding and coping with the stimuli from their internal and external environments (Eriksson and Lindström 2006). The combined use of Interpretive Description and Antonovsky's theory ensures the study remains grounded in practice while capturing the complex emotional and psychological experiences of patients, families and healthcare providers. Together, they provide a robust framework for understanding how to create a meaningful and supportive end‐of‐life environment.

### 1.3 Methods

From January to March 2023, 20 individual semi‐structured interviews were conducted (Kvale and Brinkmann 2009). The informants were selected based on their role as patient, next of a kin or health care professional. The participants were selected by DB (first author) based on her extensive experience with palliative patients and their relatives, as well as on their capacity and willingness to participate in the interviews. The interview guide was developed based on relevant topics of central literature reviews (Brereton et al. 2012; Sagha Zadeh et al. 2017). The interview questions were oriented towards eliciting open‐ended responses to acquire specific information on the perspectives of what patients, their next of a kin and health care professionals in an acute hospital find meaningful to regard when planning an end‐of‐life care room. The interview guide was tailored to the three groups, but the main focus remained consistent across them. Existing studies highlight the need to create spaces that can be adapted to the individual, feel safe and accommodate both the family and their needs. This was a key focus during the interviews. For the nursing staff, additional emphasis was placed on whether the environment met both professional requirements and workplace conditions for the staff.

### 1.4 Participants and Sample

The research sample included 20 participants: seven patients, three next of a kin and 10 health care professionals (five nurses, one health care assistants, two cleaning assistants and two doctors). The criteria for participation involved patients and their next of kin in end‐of‐life care at REDACTED, being able to take part in a face‐to‐face interview, understanding Danish and being cognitively and linguistically capable of participating in the interview and able to give confirmed consent. Inclusion criteria for health care professionals were that they had been working a minimum of 6 months in a department in an acute hospital and were experienced in giving care to patients at the end of life. This criterion was established to ensure the inclusion of practice‐based knowledge.

### 1.5 Data Generation

The interviews were conducted by the first author (DB), a palliative care nurse and experienced researcher, Ph.D.

The nurses at the ward identified potential participants. The potential participants were then included by the first author (DB) and received verbal and written information about the project. If they agreed to participate in the project, written consent was obtained. This first contact was essential to inform the participants and establish an initial relationship. The participants were informed about the interviewer's background and the reasons for conducting the research. Only the interviewer and the informant were present during the interviews. No repeat interviews were conducted and the participants did not provide feedback on the findings. All interviews were conducted face‐to‐face. The interviews with patients took place in their rooms, while those with next of kin and healthcare professionals were held in a private room on the ward. The interviews were held short to be sensitive to the seriousness of the participants' illness. In general, the duration was approximately 30 min. All interviews were audio‐recorded and transcribed verbatim with participants' consent to ensure an accurate capture of their responses and maintain the integrity of the data. No field notes were made.

The research team consisted of experienced healthcare professionals from different fields within healthcare. All researchers are female. DB is a registered nurse (RN), PhD and an experienced researcher in nursing and qualitative research. CH is a physician, LSM is a PhD‐trained anthropologist and an experienced qualitative researcher and DM is an occupational therapist (OT), senior researcher, PhD and experienced in qualitative research. DB, CH, LSM and DM contributed to idea development, fundraising and project management during the study. DB was the interviewer, and she has extensive experience in interviewing patients, next of kin and healthcare professionals, particularly in the context of palliative care. The interviews were primarily analysed by DB, LSM and DM. All authors contributed to writing and reviewing the paper. The team engaged in discussions to enhance reflexivity and critically assess potential biases, particularly those arising from their clinical or research backgrounds. They remained mindful of their assumptions and the potential impact on data collection and analysis.

### 1.6 Analysis of the Findings

Interpretive Description methodology was used for an inductive qualitative research strategy, allowing for a deep exploration of participants' experiences and preferences. Throughout the study, a practice‐based organising logic was deliberately applied to interpret and evaluate the gathered data, ensuring relevance to real‐world healthcare practices (Thorne 2016).

### 1.7 Ethical Considerations

This study adhered to the principles outlined in the Declaration of Helsinki. Prior to their participation in the interview, the patients were provided with verbal information about the project, and their informed consent was obtained. The study protocol was reviewed by the regional ethical committee of REDACTED, in accordance with Danish law (2023‐000206). However, the need for approval was waived by the committee. All relevant documents, including signed consent forms and interview transcriptions, are stored in compliance with the General Data Protection Regulation to ensure data privacy.

Given the sensitive nature of gathering information from seriously ill patients, ethical considerations were grounded in interdependent ethics, emphasising the well‐being of individuals (Løgstrup 1997). To ensure participants' comfort and privacy, interviews were conducted in quiet settings with a limited number of questions, allowing participants the option to end the interview if they felt fatigued. The study adhered to the highest ethical standards, respecting participants' rights and minimising potential harm.

### 1.8 Findings

Data analysis leads to the extraction of two themes. Participants, including patients, next of kin and healthcare professionals, unanimously emphasised the importance of aesthetic and lively physical surroundings in end‐of‐life rooms within acute hospitals. Preferences included discreetly hidden medical equipment, soothing natural elements, dimmable lighting, comfortable furniture and home‐like touches to create a comforting atmosphere. This led to the first main theme: ‘Lively physical surroundings’. The participants highlighted the importance of compassionate communication, professional competence and continuity of care. Both patients and next of kin stressed the need for consistent staff, transparent care routines and empathetic support to ensure a sense of security and dignity during the end‐of‐life phase. These findings led to the theme: ‘Human and medical competencies of the health care professionals’.

### 1.9 Lively Physical Surroundings

Among both patients, next of a kin and healthcare professionals, there is a large degree of agreement that the aesthetics of a room are of great importance in the last part of life. Patients and their next of a kin share a common desire to escape the typical hospital atmosphere in search of more lively physical surroundings. They expressed the need for certain hospital‐specific elements, such as oxygen outlets, to be discreetly concealed.

A patient said: “*Hospital‐related things like oxygen and suctions could perhaps be hidden*” and this was elaborated by another patient: “*It (the room) had to be furnished cozily—with some nice colours. I'm a bit tired of white, it's very sterile and hospital‐like”*. A next of a kin expressed it like this: “*Well, the first impression is that it's not too much like a hospital – that's probably what helps the most*.” The health care professionals described their wishes for the end‐of‐life care rooms this way: “…*there has to be pictures, flowers, plants and things like that in the room, so that it's a nice place to be*…”

Furthermore, patients and their families found solace in the incorporation of natural elements in the end‐of‐life care rooms, such as colours or illustrations inspired by the nature. This was expressed in responses like this from patients: “*I'm thinking something green or blue—I love the sea, so a sea‐blue would be beautiful to me” and “I like the sea and waves—it makes me happy and calm*”. The next of a kin also demand colours from nature: “…green colour, it's a colour that I think makes you happy—with hope and—a slightly more positive colour”. Health care professionals also mention the desire to include colours or pictures from the nature when decorating the end‐of‐life care rooms: “…*you can hang some pictures up with beautiful colours, maybe the ocean, a meadow, or something else*…”

All three participant groups placed particular emphasis on lighting. They articulated a preference for dimmable lighting, with the flexibility to adjust brightness according to individual needs. A desire for the inclusion of candles, such as LED lights, as an element to create a warm and inviting atmosphere was expressed. A patient explained; “*The ceiling light can be dimmed, but perhaps a small table lamp or some small LED lights on the table would be good, it's certainly cozy*”. This is confirmed by the next of kin: “*There is good light here, it is nice that it can be dimmed*” and by another next of kin: “…*and then maybe some candles that can create a little coziness in the evening*”. The health care professionals expressed the need for dimmed light but also that cozy lighting that makes it more homely.

In addition to lighting, respondents conveyed a yearning for cosines and homeliness within the hospital room. This extended beyond candles to encompass soft, soothing colours, wall art, cushions, carpets and tablecloths. Notably, the presence of potted plants was a recurring theme. The patients expressed a desire for the living rooms to look like home: “… *perhaps some plants, some light, those battery‐powered candles are nice. Some shelves where they can place personal items, so it feels like home*”. The next of kin expressed it this way: “…*it could also be some flowers—what are those called—faux flowers, it also gives some life and some security of one kind or another*”.

A healthcare professional further emphasised the importance of aesthetics, mentioning, “…*a nice bedspread and some pillows, so it doesn't look like a hospital bed*”.

The prevailing sentiment in our study highlights the crucial role that aesthetics play in the well‐being and comfort of patients and their families during the final stages of life. Creating an environment that accommodates these aesthetic preferences contributes to a more compassionate and comforting healthcare experience. The patients and their next of kin emphasise the need for nice and comfortable furniture, space and a place for relaxation. A patient expresses a wish of some kind of wardrobe: “*Yes, with a mirror and where you could put your shoes, hang your jacket, that welcomed you—and I also think it's nice that there is a sofa, for example, where there are children, where there are cushions and blankets, and here I have to play—it could well be a few teddy bears—there must be room for everyone—it doesn't have to be so grown‐up*”. The next of a kin demand comfortable furniture: “… *two good chairs would be nice—we would like to sit here by the window, and then we can sit together and eat, talk or watch television”* another next of kin added the following: “Yes, it is nice to sit and have a conversation and relax—it is also demanding to be very present for a long time—and it is hard to sit for 5 h in a bad chair”. The need for comfortable furniture is expressed by a health care professional this way: “*So that's what I think when I see the next to kin, it has to be comfortable for them because, they've neglected themselves for a long time and they keep on doing it the same when they come in here—so they don't feel, what they need*”.

The patients also relate to sound, in this context regarding the transport of food carts in the corridor: “I was absolutely frantic because of the noise. It's like having a shipyard as a neighbour, there was such unrest”.

All the professional groups emphasise the necessary of space for performing their work, and easy access to the remedies that are used frequently. The cleaning assistants express the importance of being able to keep the end‐of‐life rooms clean: “*Sofas and chairs should have high legs, because it's in there underneath, that's where the dust settles, so we'd like to be able to get under there with a mop…”*. The nurses emphasise the importance of having both good working environment conditions but also space for next of a kin: “… *we have to come in and help to turn the patient around, or just help a little with that, so there must also be room for us, but at the same time there must also be room for the next to kin, so it's a bit of a balancing act*”. A doctor explains that they do not need much space with the palliative patients “… *if they can sit up in bed, and we can maybe just get them out of bed, then that's fine, but I don't even think we'll do that”*. With that said, the health care professionals also emphasised the need for homely and/lively physical surroundings to support their palliative work.

As part of creating lively physical surroundings food and gastronomic is important for the patients. Both in relation to holding on to life, but also in relation to quality of life. A patient expresses the importance of being able to eat a meal with his next of a kin: “… then we should be able to sit and eat together—I probably don't eat that much, but I like to sit there with a glass of water or juice and hear what they are talking about”. Another patient expresses that well prepared food is important to him also in the last period of life: “*Well, it means a lot that it's good food. And I know it's good food. That's why I chose the soup”*.

The lively physical surroundings are of great importance at the end of life, but yet the medical care and interactions with healthcare professionals are equally crucial.

### 1.10 Human and Medical Competencies of the Health Care Professionals

Patients and next of kin express unequivocally that the human skills in terms of being able to communicate and understand the needs of both the patient and the next of kin are very important. “It *is their kindness, all the time, always kind, always smiling and always time, I think*”

Providing care to end‐of‐life patients often requires something special from the staff, both in terms of human and professional skills. A patient describes it this way: “*There is a difference in the quality of the nurses. There are some who can figure it out and some who can't*”.

Both patients and their next of kin underscore the importance of feeling welcomed and acknowledged in a situation that is highly stressful for everyone involved. A close next of kin articulates this sentiment, stating, “It's about life or death, and you're under more pressure than you've ever been in your life.”

The patients express that it creates insecurity if they have doubts about the staff's professional competence. One of the skills is having an overview of what needs to be done when: “*The good staff, they are the ones who have the overview. There is someone who does not have the overview. You have to pull it there four times so that they can find out when the drop has to come out and so on*”.

It is crucial that patients and their next of kin do not perceive themselves as inconveniences. One individual shared an experience: “… we pressed the call button when an issue arose, and then we could hear them saying in the corridor: ‘What do they want now? I've just been in there,’ and then another nurse takes her place, sending someone else because she didn't want to bother herself.”

The doctor's express positivity regarding the new end‐of‐life rooms but emphasise that their primary focus in selecting procedures is to perform surgeries. One doctor clarifies, “We want our patients to feel good, don't get it wrong, but that's not our primary concern. It's not palliation; it's operating on our patients.”

Patients and next of kin emphasise the importance of staff—both doctors and nurses—being extremely competent in terms of knowledge of medication and not least in relation to when the medication should be given. It is also important that there is agreement between the statements coming from the various health professionals. A patient gives this example: “… *then I get penicillin in the evening at 10 o'clock, hung up, and then they have to switch over, and then they have to give saline at the end—and then I can wake up at 5 o'clock and nothing has progressed—and I have a system that is open. I might get a little worried about that—imagine if there was blood poisoning—that's my own concern—and sometimes they come and ask how many times you flush your drain—I do that twice—well, okay—so does that uneasiness come—is this the right treatment I'm getting—and I had 5 blood clots in my lungs, and for example today I haven't been given a blood thinner, I think they forgot that, so I have to ask for it*…”.

Medication and symptom relief is essential and both doctors and nurses require the need for support, either guidelines or framework prescriptions. A doctor proposes framework prescriptions: “*just the basics, because if you can just pull it up, then we've come a long way, and then you can save a lot of extra consultations”*, this is confirmed by a nurse: “Yes, you could make such a framework prescription, not everyone fits into it, but then you can start up, and then you can increase it or change it—it would be a huge help if they have it to assume”. Some of the health care professionals request the possibility of bystander training in relation to how the difficult conversations can be handled with patients and next of kin in this last difficult phase of life “*Yes, but maybe you could do some short courses here, maybe in the afternoon, for a couple of hours, where we could hear something about symptoms, but also about how to talk to the patients, because it can be a bit difficult…”*

Furthermore, both patients and their next of kin express a desire for transparency regarding activities, like when ward rounds are taking place. A next of kin says: “*for example, when there is a round—I know that you can never plan for that, but if there was a structure in it, that you knew a little more—it is between 9 and 9.30—so you could come, because it concerns a lot of people—and it's a lot of people who want to be there for those doctor's appointments—and it's a very big gap if it's from 9 o'clock and 12 o'clock—there is not that much planning in it*.”

Patients, their next of kin and healthcare professionals, consistently emphasise the paramount importance of ensuring that patients receiving palliative care have the opportunity to interact with the same caregiving staff whenever feasible. A next to kin explains this way why it is important to have the same contact persons: “*it could be nice if it was the same doctor you had, that it wasn't always a new one—then a new doctor comes, then he has a different conclusion, and then another doctor comes, and then he has another conclusion—that is, the thing about it being the same or two doctors you had to deal with who know him, I would like that*”. The wish also applies to having the same nurse: “…it would be nice to have someone who was our nurse, but we can't demand for that…”. The health care professionals are also aware of the desire for a permanent contact person, but this is not always possible: “*Yes, we do now, as far as possible, yes. Now I've been here over the weekend, so I want to have the same group as much as possible. It's not always possible to do it, but I hope with that living room that it makes it possible that you can, so that you don't have to look for a bell to see if it's your own, that's not good.”*

A patient also emphasises the need for calm, and in some cases also with regard to the next of kin: “*I think it is important to give peace of mind as next of kin—because I think it has been hard here, where everyone has been here—it has been my siblings, my children, my parents and my parents‐in‐law and my sisters‐in‐law and brothers‐in‐law—that is I've probably had a hard time with that and it's tired me out a lot*…”. It is important that the staff is aware of supporting the patient in needing peace, and that it can also be too much to be surrounded by the next of kin for too long at a time.

When planning a new end‐of‐life room in a department of surgery in an acute hospital it is essential to involve the healthcare professionals. Basically, the health care professionals did not feel oriented about which patients should be admitted to the palliative care rooms and who should lie in the rooms intended for surgical patients. A nurse says: “…I think that some guidelines should have been found for what kind of patients are to lie in the end‐of‐life care beds…”. Palliative patients have special medical needs, that are not routinely addressed in a surgical department, as well as extended needs in human relation with the health care staff and this complexity requires to be articulated and made visible in the department's daily routines.

## 2 Discussion

This qualitative study highlights the importance of focusing on the lively physical surroundings and human and medical competencies of the health care professionals when end‐of‐life care rooms are set up on a non‐palliative ward, where there are requirements for hygiene and an appropriate working environment. The informants agree about the importance of furnishing the end‐of‐life care rooms in a way so that the hospital atmosphere is avoided, which confirms more existing studies (McLaughlan et al. 2022; McLaughlan and Kirby 2021; Wong et al. 2023).

A substantial body of empirical literature explores the correlation between nature exposure and health outcomes, particularly within palliative care contexts (Jimenez et al. 2021; Sagha Zadeh et al. 2017). Extensive evidence supports the notion that exposure to natural environments positively influences sleep patterns (Shin et al. 2020). Moreover, well‐documented findings indicate a reduction in cortisol levels among individuals exposed to natural settings (Song et al. 2016). Given the significance of sleep quality and cortisol regulation in end‐of‐life scenarios, these factors hold paramount importance for both patients and their next of kin. Additionally, the positive impact extends to the enhancement of psychological well‐being among patients, their next of kin and healthcare professionals alike (Sagha Zadeh et al. 2017). The present study suggests implementing nature when planning a new end‐of‐life room in a hospital setting, confirming existing studies where both patients, next of kin and healthcare professionals recommend this (Brereton et al. 2012; Gardiner et al. 2011; Wong et al. 2023).

The participants agreed about the importance of the lighting within the room. It is well‐known that the light intensity and colour can affect patients' and staff's mood and health. This finding is in line with existing literature demonstrating that light intensity and colour significantly influence mood, well‐being and overall health outcomes for both patients and healthcare staff (Wong et al. 2023). In the current study, participants advocated for the adoption of soft lighting, specifically endorsing candles with LED illumination. This choice was motivated by the potential of such lighting to cultivate a home‐like and comforting atmosphere. The expressed desire to imbue end‐of‐life environments with a sense of homeliness, in contrast to the typical hospital setting, emerged as a central theme and aligns with findings from a review by Wong et al. (Wong et al. 2023). The participants in the present study did not define homeliness, but used words such as: nice colours, pictures, flowers, plants, cushions, carpets and tablecloths—words that are comparable to other studies describing the need for homeliness during the last days of life (Fleming et al. 2015; Timmermann et al. 2015). The patients and healthcare professionals suggested hiding medical equipment in the room; while it is not necessary in end‐of‐life care, these statements correlate with a study by Zadeh et al. (Sagha Zadeh et al. 2017).

The meal itself, but also the possibility to eat a meal with the family, is mentioned as important, despite the appetite not existing. The meal facilitates interaction with the family and the feeling of everyday life—a feeling of high value for the patients (Buchwald et al. 2024; Gardiner et al. 2011).

The physical environment is of great importance. But the informants' statements also support that the physical environment is a supporting factor on the human and professional capabilities of the staff when providing qualified treatment to dying patients. Nurses specialising in end‐of‐life care assume diverse responsibilities, encompassing medication administration and personal care. Additionally, they play an important role in advocating for patients and their families, providing support through the emotional challenges of fear, anxiety and grief during the final days of life. The informants underscore the importance of both professional competencies in medication administration and personal qualities during severe illness, particularly when they and their next of kin are under heightened pressure. The patients and their next of kin do not define their needs according to communication with nurses and doctors at end‐of‐life. Research emphasises the crucial role of effective communication training (Löfmark et al. 2007; Long et al. 2019; Schultz and Bar‐Sela 2013). The participants appreciated meeting competent professionals who also were caring human beings. Only a few studies explore how patients and next of kin experience the relationship with health care professionals in end‐of‐life care, but personal competencies such as friendliness, cheerfulness and good manners are important under stressful circumstances during the last days of life (Gourdji et al. 2009; Lind et al. 2012; Spichiger 2010).

The doctors request guidelines for treatment and medication for these end‐of‐life patients, who are a minority of the patients they treat in daily practice. There are national and local guidelines, but more detailed guidelines are requested (The National Board of Health 2017). All three groups of informants prefer a continuity of care from both nurses and doctors in end‐of‐life care, which confirms the limited existing knowledge about this theme (den Herder‐van der Eerden et al. 2017).

### 2.1 Strengths and Limitations

A key strength of this study is the successful inclusion of patients, next of kin and healthcare professionals, ensuring that all perspectives are represented. Unlike many studies that focus on hospices or palliative care units, this study involves participants from a surgical ward. By incorporating all three groups within the setting where the findings will be applied, the study enhances the transferability of the knowledge directly to clinical practice. With this said, a limitation is the limited number of participants, particularly among patients and their next of kin. This limitation is exacerbated by the brevity of interviews, a constraint directly tied to the vulnerability of this specific informant group, impacting their ability and energy to participate in‐depth interviews. The involvement of the first author (DB) in conducting the interviews presents both a strength and a limitation. As a nurse in the specialised palliative team, her familiarity with some informants may have influenced her with preconceived notions of their needs. However, this familiarity also afforded her a profound understanding of the unique requirements of these patients and their families. Another limitation is that the transcripts were not returned to the participants due to their life circumstances. The study's strength lies in its multidisciplinary approach, offering diverse perspectives that fostered critical methodological discussions.

## 3 Conclusion

Patients, their families and healthcare professionals involved in this study underscore the desire for future end‐of‐life rooms to exude a homely ambiance, distinctly detached from the clinical setting. Decorative elements, including carefully chosen colours and nature‐themed illustrations, play a crucial role. Emphasising the significance of light, participants advocate for a warm atmosphere achieved through floral arrangements, cushions and blankets. The imperative for accommodating entire families and facilitating communal activities, such as shared meals, is underscored. Balancing these comfort considerations, there is a consensus on maintaining cleanliness and ensuring optimal working conditions for staff. In light of contemporary pressures on patients and their families, participants highlight the need for staff understanding and responsiveness, emphasising transparency and the establishment of defined living spaces. Guidelines are recommended to uphold a consistently high professional standard, even for staff not specialising in palliative care. Addressing these multifaceted needs is vital for enhancing the end‐of‐life experience for all involved.

### 3.1 Recommendations for Future Results/Implications for Practice

In an acute hospital setting, the design of end‐of‐life rooms is recommended to feature a homely and non‐clinical aesthetic, incorporating elements inspired by nature using colours, plants and soft lighting. Furnishings such as pillows and a bedspread contribute to creating a comforting atmosphere.

The room should facilitate interaction between patients and their next of kin, providing space for shared activities such as meals, and overall, should be a welcoming environment for extended stays.

Patients and their families emphasise the importance of both human and professional capabilities of healthcare professionals. Furthermore, there is an expressed need for guidelines, especially concerning medication administration, to ensure a high standard of professional and effective treatment.

Implementation of end‐of‐life beds on a surgical ward requires that a strong collaboration between the interdisciplinary specialties and specialist groups has been described and initiated.

## Author Contributions

D.B., C.H., L.S.M. and D.M. contributed to idea development, fundraising and project management during the study. D.B. was the interviewer, and she has extensive experience in interviewing patients, next of kin and healthcare professionals, particularly in the context of palliative care. The interviews were primarily analysed by D.B., L.S.M. and D.M. All authors contributed to writing and reviewing the paper.

## Conflicts of Interest

The authors declare no conflicts of interest.

## Data Availability

The data that support the findings of this study are available from the corresponding author upon reasonable request.
